# A nomogram to predict the absence of clinically significant prostate cancer in males with negative MRI

**DOI:** 10.1590/S1677-5538.IBJU.2024.0084

**Published:** 2024-03-10

**Authors:** Masatomo Kaneko, Atsuko Fujihara, Tsuyoshi Iwata, Lorenzo Storino Ramacciotti, Suzanne L. Palmer, Masakatsu Oishi, Manju Aron, Giovanni E. Cacciamani, Vinay Duddalwar, Go Horiguchi, Satoshi Teramukai, Osamu Ukimura, Inderbir S. Gill, Andre Luis Abreu

**Affiliations:** 1 University of Southern California USC Institute of Urology and Catherine & Joseph Aresty Center for Image-Guided Surgery Los Angeles California USA USC Institute of Urology and Catherine & Joseph Aresty, Center for Image-Guided Surgery, Focal Therapy, and Artificial Intelligence for Prostate Cancer, Keck School of Medicine, University of Southern California, Los Angeles, California, USA; 2 Kyoto Prefectural University of Medicine Graduate School of Medical Science Department of Urology Kyoto Japan Department of Urology, Graduate School of Medical Science, Kyoto Prefectural University of Medicine, Kyoto, Japan; 3 University of Southern California Keck School of Medicine Department of Radiology Los Angeles California USA Department of Radiology, Keck School of Medicine, University of Southern California, Los Angeles, California, USA; 4 University of Southern California Keck School of Medicine Department of Pathology Los Angeles California USA Department of Pathology, Keck School of Medicine, University of Southern California, Los Angeles, California, USA; 5 University Hospital The Clinical and Translational Research Center Division of Data Science Kyoto Japan Division of Data Science, The Clinical and Translational Research Center, University Hospital, Kyoto Prefectural University of Medicine, Kyoto, Japan; 6 Kyoto Prefectural University of Medicine Graduate School of Medical Science Department of Biostatistics Kyoto Japan Department of Biostatistics, Graduate School of Medical Science, Kyoto Prefectural University of Medicine, Kyoto, Japan

**Keywords:** Prostatic Neoplasms, Multiparametric Magnetic Resonance Imaging, Biopsy

## Abstract

**Purpose:**

To create a nomogram to predict the absence of clinically significant prostate cancer (CSPCa) in males with non-suspicion multiparametric magnetic resonance imaging (mpMRI) undergoing prostate biopsy (PBx).

**Materials and Methods:**

We identified consecutive patients who underwent 3T mpMRI followed by PBx for suspicion of PCa or surveillance follow-up. All patients had Prostate Imaging Reporting and Data System score 1-2 (negative mpMRI). CSPCa was defined as Grade Group ≥2. Multivariate logistic regression analysis was performed via backward elimination. Discrimination was evaluated with area under the receiver operating characteristic (AUROC). Internal validation with 1,000x bootstrapping for estimating the optimism corrected AUROC.

**Results:**

Total 327 patients met inclusion criteria. The median (IQR) age and PSA density (PSAD) were 64 years (58-70) and 0.10 ng/mL2 (0.07-0.15), respectively. Biopsy history was as follows: 117 (36%) males were PBx-naive, 130 (40%) had previous negative PBx and 80 (24%) had previous positive PBx. The majority were White (65%); 6% of males self-reported Black. Overall, 44 (13%) patients were diagnosed with CSPCa on PBx. Black race, history of previous negative PBx and PSAD ≥0.15ng/mL2 were independent predictors for CSPCa on PBx and were included in the nomogram. The AUROC of the nomogram was 0.78 and the optimism corrected AUROC was 0.75.

**Conclusions:**

Our nomogram facilitates evaluating individual probability of CSPCa on PBx in males with PIRADS 1-2 mpMRI and may be used to identify those in whom PBx may be safely avoided. Black males have increased risk of CSPCa on PBx, even in the setting of PIRADS 1-2 mpMRI

## INTRODUCTION

Multiparametric magnetic resonance imaging (mpMRI) is recommended by guidelines for patients with a suspicion for prostate cancer (PCa) before prostate biopsy (PBx). There is a clear recommendation to biopsy all males with Prostate Imaging-Reporting and Data System (PIRADS) 3-5 mpMRI. However, the recommendation to biopsy those with PIRADS 1-2 mpMRI (non-suspicious/negative), including those on active surveillance (AS), is not well defined and should be considered on a case-by-case basis, sharing the decision with the patient (1, 2).

Approximately 25% of clinically significant cancer (CSPCa) can be missed in the setting of PIRADS 1-2 mpMRI (3). On the other hand, performing PBx on patients with PIRADS 1-2 mpMRI can increase biopsy-related morbidity and overdiagnosis by detecting clinically insignificant prostate cancer (CIPCa). Therefore, more precise methods to evaluate individual risk of CSPCa are necessary. The decision to biopsy a patient with PIRADS 1-2 mpMRI is complex and multifactorial, including patient’s race, biopsy history, prostatic antigen (PSA) density (PSAD) and others (4-9). We hypothesized that a nomogram incorporating clinical variables in males with PIRADS 1-2 on mpMRI may facilitate personalized decision making whether to perform prostate biopsy.

The objective of the current study is to create a nomogram to predict the absence of CSPCa in males with PIRADS 1-2 mpMRI undergoing PBx.

## MATERIALS AND METHODS

### Study design and population

The current study was approved by our Institutional Review Board (IRB No. HS-13-00663). We identified consecutive patients who underwent PBx at University of Southern California (USC), from September 2011 to August 2019, from our prospectively maintained PBx database. The inclusion criteria were: Males with i) suspicion for PCa by elevated or rising PSA, abnormal digital rectal examination (DRE) or those on AS for PCa; ii) 3T mpMRI within 6 months before PBx; iii) PIRADS 1-2 mpMRI (negative mpMRI); iv) extended sextant systematic PBx. Exclusion criteria were: i) mpMRI that did not meet PIRADS standards (version 1.0 for before 2015, 2.0 for 2015 to April 2019, and 2.1 for after May 2019); ii) any prior treatment for PCa; iii) prior surgical therapy for enlargement of the prostate or lower urinary symptoms; iv) mpMRI with inadequate quality (i.e. 1.5T or significant artifact); v) mpMRI performed longer than 6 months before biopsy.

All cases had no prostate cancer suspicious findings on mpMRI (PIRADS 1-2). All patients with PIRADS 1-2 mpMRI routinely underwent extended sextant systematic PBx as our institutional daily practice (6, 10).

### MRI acquisition and Imaging interpretation

The exams were performed on a 3T MR-750 MR scanner (General Electric, USA) with a 16-channel phased-array surface coil. Sequences included small field of view axial, coronal, and sagittal T2-weighted (T2W), diffusion-weighted imaging (DWI) using b100, b800 and b1400, apparent diffusion coefficient (ADC) map, and dynamic contrast-enhanced (DCE) during the intravenous injection of 0.2mL/kg gadobenate dimeglubine (MultiHance, Bracco Diagnostics, Germany) at 3 mL/s (10). mpMRI was acquired and interpreted based on PIRADS version 1.0 (before 2015), 2.0 (after 2015) or 2.1 (after May 2019) according to the current version at time of biopsy (11-13). MRIs performed outside institution were accepted if they met PIRADS standards and inclusion/exclusion criteria. Images were evaluated by experienced radiologists and reports were further reviewed by an experienced radiologist (SP) with more than 15 years reading mpMRI prostate to confirm these images had no cancer suspicious lesion (6, 10).

### Prostate biopsy protocol

Transrectal ultrasound (TRUS)-guided systematic extended sextant 12-core PBx were performed transrectally, using the Koelis ® system (Koelis ®, Grenoble, France) and 18G needle-biopsy, under local anesthesia by two experienced urologists at USC (OU and ALA), as previously described (6, 10, 14-16). The same template was applied to all patients (6, 10).

### Definitions and endpoint

The endpoint is the absence of CSPCa on PBx. CSPCa was defined as International Society of Urological Pathology (ISUP) Grade Group (GG) 2 or greater (6, 10, 14, 15, 17). CIPCa was defined as ISUP GG 1. Prostate volume (PV) was measured on mpMRI using ellipsoid formula (PV = height x width x length x 0.52). Patient’s race was determined as self-assessed by the patients according to National Institutes of Health guidelines (18). PSAD was evaluated as continuous variable and as dichotomized variable specifically using a cut off of PSAD ≥0.15ng/mL2, as previously defined (6, 14). Patients on AS, were considered as having a history of prior positive biopsy.

### Statistical Analysis

Patient characteristics were analyzed descriptively. The patients were divided into two cohorts according to biopsy histology, including: benign or CIPCa versus CSPCa cohort. The Wilcoxon rank sum test was used for continuous variables and the Fisher exact test was used for categorical variables. Univariate logistic regression analysis was performed using clinical and demographic parameters. Multivariate logistic regression analysis was performed using the predictors systematically selected via stepwise backward elimination methods. The exit criteria were centered p-value threshold of 0.25. The model performance was assessed with respect to discrimination and calibration. Discrimination was evaluated with area under the receiver operating characteristic (AUROC). Internal validation with 1,000x bootstrapping for estimating the optimism corrected AUROC (19). Calibration was examined with calibration plots and the Hosmer-Lemeshow test (20). The nomogram was generated based on a multivariate logistic regression model. The effect with the highest regression coefficient was assigned 100 points on the scale, and the other variables were assigned points proportional to their effect size regardless of statistical significance (21). Statistical analyses were performed using SAS version 9.4 (SAS Institute Inc., Cary, NC, USA) and RStudio version 1.2 (RStudio, Inc., USA) with the rms library. A two-sided p-value <0.05 was considered significant.

## RESULTS

**Figure 1:**
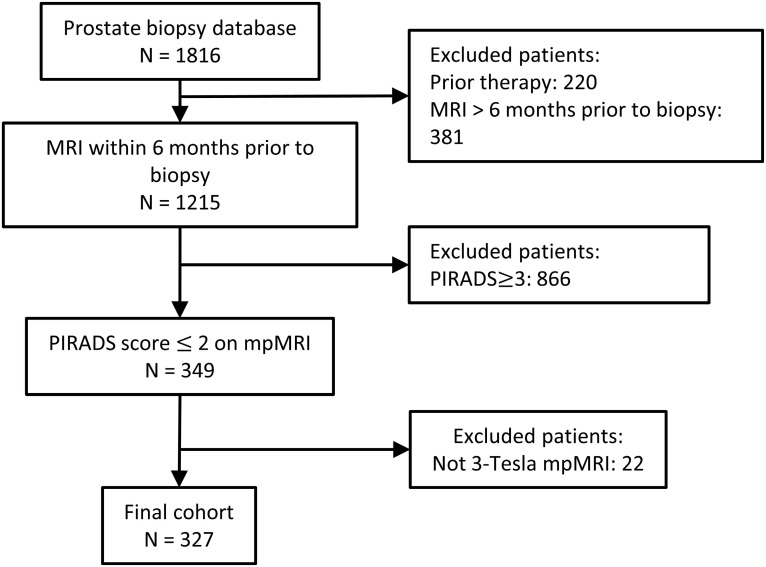
Study cohort flowchart.

A total of 327 patients met inclusion criteria (Figure-1). Demographics, clinical and pathological characteristics are shown in Table-1. The median (IQR) age, PSA, PV, PSAD, number of positive cores per patients, maximum cancer core length and percent were 64 years (58-70), 6.0ng/mL (4.4-8.4), 59mL (40-86), 0.10ng/mL2 (0.068-0.15), 0 (0-1), 4mm (1-6) and 15% (5-40), respectively. Majority of the patients were White (65%); 6% self-reported Black. Abnormal DRE was found in 45 (14%) and 82 (25%) had family history of PCa. Prostate biopsy history was as follows: 117 (36%) were PBx naive, 130 (40%) had prior negative PBx, and 80 (24%) on AS had prior positive PBx (73 GG1, 6 GG2, and 1 GG3). The median (IQR) number of prior biopsies (including those on AS and those with negative biopsy) was 1 (1-2); and the time from last biopsy to current biopsy was 16 (7-37) months. For patients on AS (N=80), the last surveillance biopsy showed PCa in 71 (89%) males and was benign in 9 (11%).

**Table 1:** Demographics of patients with negative MRI undergoing systematic prostate biopsy according to histology findings.

		All	Benign or CIPCa	CSPCa	
Variables		Median (IQR) or N (%)	Median (IQR) or N (%)	Median (IQR) or N (%)	p-value
No. of patients		327 (100)	283 (87)	44 (13)	–
Age, years		64 (58-70)	64 (58-70)	66 (59-70)	0.62
PSA, ng/mL		6.0 (4.4-8.4)	6.0 (4.4-8.4)	6.1 (4.6-8.5)	0.70
Prostate volume, mL		59 (40-86)	60 (42-90)	37 (27-62)	< 0.001
PSA density, ng/mL^2^		0.10 (0.07-0.15)	0.10 (0.07-0.14)	0.14 (0.09-0.22)	< 0.001
**Race**					0.004
	Black	19 (6)	11 (4)	8 (18)	
	White	214 (65)	189 (67)	25 (57)	
	Asian	29 (9)	24 (9)	5 (11)	
	Others	13 (4)	12 (4)	1(2)	
	Unknown†	52 (16)	47 (17)	5 (11)	
Suspicion DRE		45 (14)	41 (14)	4 (9)	0.48
**Prior prostate biopsy status**					0.01
	Naive	117 (36)	101 (36)	16 (36)	
	Prior negative	130 (40)	120 (42)	10 (23)	
	Prior positive	80 (24)	62 (22)	18 (41)	
Family history of prostate cancer		82 (25)	71 (26)	11 (26)	1.0
PIRADS version 2		203 (62)	180 (64)	23 (52)	0.18
No. of positive cores		0 (0-1)	0 (0-0)	5 (3-7)	< 0.001
**ISUP Grade Group**					< 0.001
	0	213 (65)	213 (75)	0 (0)	
	1	70 (21)	70 (25)	0 (0)	
	2	29 (9)	0 (0)	29 (66)	
	3	11 (3)	0 (0)	11 (25)	
	4	2 (1)	0 (0)	2 (5)	
	5	2 (1)	0 (0)	2 (5)	
Maximum cancer core length, mm		4 (1-6)	1.5 (1-5)	6 (4-8)	< 0.001
Maximum cancer core, %		15 (5-40)	9 (5-20)	30 (25-50)	< 0.001

† = Patients that did not self-report their race;

DRE = digital rectal examination; MRI = magnetic resonance image; IQR = interquartile range; PSA = prostate specific antigen; No. = number; CIPCa, clinically insignificant prostate cancer; CSPCa = clinically significant prostate cancer; PIRADS = Prostate Imaging-Reporting and Data System; ISUP = International Society of Urological Pathology.

Overall, 44 (13%) patients were diagnosed with CSPCa on PBx. Comparison between benign or CIPCa group versus CSPCa group showed that PV, PSAD, race, prior PBx status, number of positive cores, maximum cancer core length and percent were significantly different between the two groups (Table-1).

### Building the Nomogram

**Figure 2:**
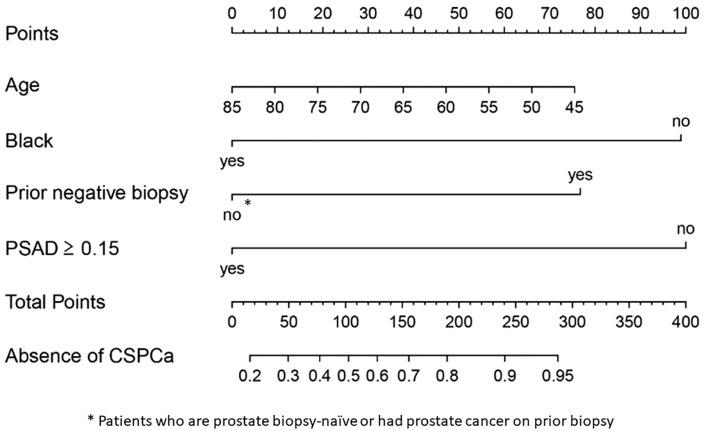
Nomogram to predict the absence of clinically significant prostate cancer in patients with negative multiparametric MRI.

**Figure 3:**
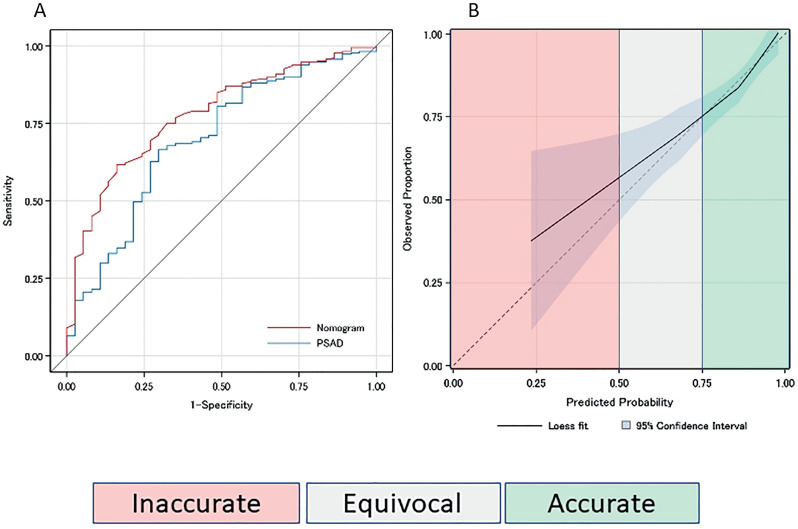
Receiver operating characteristic curve analysis of the model in predicting the absence of clinically significant prostate cancer in males with negative multiparametric MRI of the prostate and calibration plot of the nomogram.

Univariate logistic regression analysis showed that Black race, smaller PV, PSAD≥0.15ng/mL2 and prior negative PBx status were significant predictors for CSPCa on PBx. PV and PSAD were both significant predictors for CSPCa on PBx, however, because of collinearity between the two variables, only PSAD was selected for multivariate analysis model. Black race, history of previous negative PBx, and PSAD≥0.15ng/mL2 were independent predictors for CSPCa on PBx, and therefore were included into the nomogram (Table-2). After stepwise selection, age was kept in the nomogram due to the clinical relevance. A nomogram to predict absence of CSPCa was then built using age and the independent predictors variables on multivariable analysis, as follows: age (OR 0.97, p=0.23), Black race (OR 0.21, p = 0.005), history of previous negative PBx (OR 3.40, p = 0.005), and PSAD ≥0.15ng/mL2 (OR 0.20, p < 0.005) (Figure-2). The nomogram was internally validated with 1,000x bootstrapping, which provided the optimism corrected AUROC was 0.75 (Figure-3A). The Hosmer-Lemeshow test showed the model was well calibrated (p = 0.79) (Figure-3B).

**Table 2:** Univariate and multivariate logistic regression analysis to predict the absence of clinically significant prostate cancer in patients with negative MRI.

		Univariate			Multivariate		
Variables		OR	CI (95%)	p-value	OR	CI (95%)	p-value
Age (years)		0.99	0.95-1.03	0.64	0.97	0.92-1.02	0.23
**Race** †							
	Black	0.19	0.07-0.51	0.001	0.21	0.07-0.62	0.005
	White	2.25	1.09-4.66	0.03			
	Asian	0.77	0.28-2.15	0.62			
	Other	2.04	0.26-16.11	0.50			
PSA (ng/mL)		1.00	0.94-1.07	0.91			
Prostate volume (mL)		1.02	1.01-1.04	<0.001			
PSAD ≥ 0.15 (ng/mL^2^)		0.28	0.14-0.56	<0.001	0.20	0.09-0.44	<0.001
**Prostate biopsy status**							
	Naïve	0.97	0.50-1.88	0.93			
	Prior negative	2.50	1.19-5.26	0.02	3.40	1.44-8.03	0.005
	Prior positive	0.41	0.21-0.79	0.008			
	Suspicion DRE	1.69	0.58-4.99	0.34			
Family history PCa		1.04	0.50-2.17	0.92			
MRI location (USC vs elsewhere)		1.66	0.68-4.05	0.29			
PIRADS version (2 vs 1)		1.60	0.84-3.02	0.15			

† = Patients who did not self-report their race were removed from these analyses;

DRE = digital rectal examination; PSA = prostate specific antigen; PSAD = PSA density; PCa = prostate cancer; PIRADS = Prostate Imaging-Reporting and Data System; USC = University of Southern California; IQR = interquartile range; CI = confidence interval; OR = odds ratio.

The nomogram combines the variables age, Black race, prior negative biopsy status, and PSAD ≥ 0.15ng/mL2. In order to use the nomogram, first, locate the value of the variable on its axis. Second, draw a line straight upwards to the "Points" axis and determine the number of points for the variable. After repeating this procedure for all four variables, sum up the total points. Finally, locate the "Total Points" axis according to the total points, and draw a line downward to the "Absence of CSPCa" axis to determine the probability of the absence of CSPCa.

A. Red line indicates ROC curve of the model selected via backward elimination methods. AUROC of the model was 0.78 and the optimism corrected AUROC was 0.75. Blue line indicates ROC curve of PSAD as a continuous variable for reference. AUROC of PSAD was 0.697.

B. Calibration plot of the nomogram.

Dashed line indicates ideal reference line where predicted probability would match observed proportions. Solid line represent Loess fit model based on predicted probability using the nomogram. The graph is color-coded according to the accuracy of the predicted probability (PP): inaccurate (PP <0.5) in red; equivocal (0.5≤ PP <0.75) in gray; accurate (PP≥0.75) in green.

AUROC, area under the receiver operating characteristic; MRI, magnetic resonance image; PP, predicted probability; PSAD, prostate specific antigen density; ROC, receiver operating characteristics.

### Nomogram interpretation

**Figure 4:**
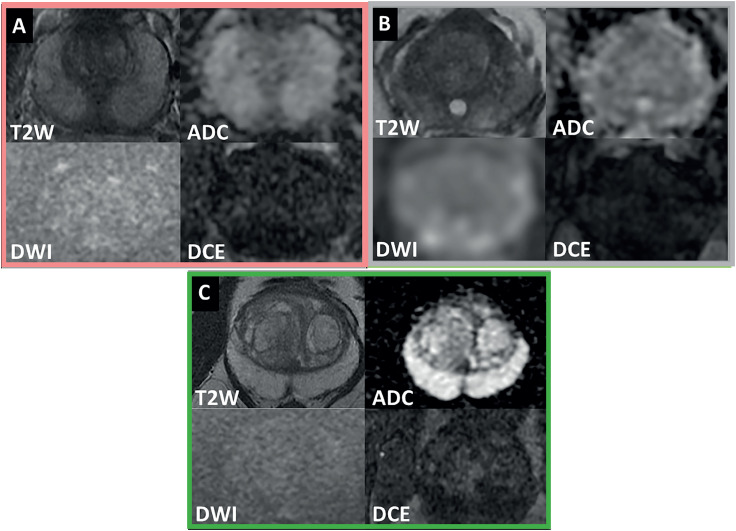
Nomogram application: Index cases

The high predicted probability (PP) ≥0.75 part of the nomogram matched the actual observed count, with a majority of males falling into this risk-assessment interval, therefore the nomogram is accurate and prostate biopsy may be safely avoided (Table-3; Table-S1; Figure-3B; Figure-4). For the prediction probability between 0.5 to 0.75, the confidence interval becomes wider with smaller number of patients and tendency for underestimation. For this PP interval (0.5≤ PP <0.75), the nomogram is equivocal and prostate biopsy might be considered. For the PP interval up to 0.5, the confidence interval is wide and there is a small number of patients, and the nomogram is not precise; therefore, prostate biopsy should be considered. When physicians accept predicted probability 0.75 as a cutoff for omitting systematic biopsy, 88.8% of systematic PBx for patients with negative MRI can be safely omitted, at the cost of missing 9.6% of CSPCa. On the other hand, if no males with negative MRI undergo biopsy, additional 11.2% of systematic PBx can be omitted, at the cost of missing 13.7% of CSPCa.

**Table 3:** Subdivided performances of the nomogram based on different cutoffs to predict the absence of CSPCa on prostate biopsy in males with negative MRI.

Cutoff PP	Sensitivity	Specificity	NPV	PPV	Safely omittable PBx	Missed CSPCa
0.95	0.318	0.973	0.185	0.987	0.317	0.013
0.90	0.549	0.865	0.234	0.962	0.549	0.037
0.85	0.815	0.541	0.317	0.918	0.815	0.082
0.80	0.871	0.432	0.348	0.906	0.871	0.093
0.75	0.888	0.405	0.366	0.904	0.888	0.096
0.70	0.893	0.378	0.359	0.900	0.892	0.099
0.65	0.936	0.270	0.400	0.890	0.935	0.110
0.60	0.961	0.135	0.357	0.875	0.961	0.125
0.55	0.996	0.081	0.750	0.872	0.995	0.127
0.50	0.996	0.027	0.500	0.866	0.995	0.134
0.45	0.996	0.027	0.500	0.866	0.995	0.134
0.40	0.996	0.027	0.500	0.866	0.995	0.134
0.35	0.996	0.027	0.500	0.866	0.995	0.134
0.30	0.996	0	0	0.862	0.995	0.137
0.25	1	0	–	0.863	1	0.137
0.20	1	0	–	0.863	1	0.137
0.15	1	0	–	0.863	1	0.137
0.10	1	0	–	0.863	1	0.137
0.05	1	0	–	0.863	1	0.137

CSPCa = Clinically significant prostate cancer; NPV = negative predictive value; PBx = prostate biopsy; PP = predicted probability; PPV = positive predictive value.

Safely omittable PBx was defined as dividing true positive cases by no CSPCa case number.

Missed CSPCa was defined as dividing false positive by case number above cutoff PP.

**Table S1:** Distribution of predicted probability and observed absence of CSPCa on prostate biopsy in males with negative MRI.

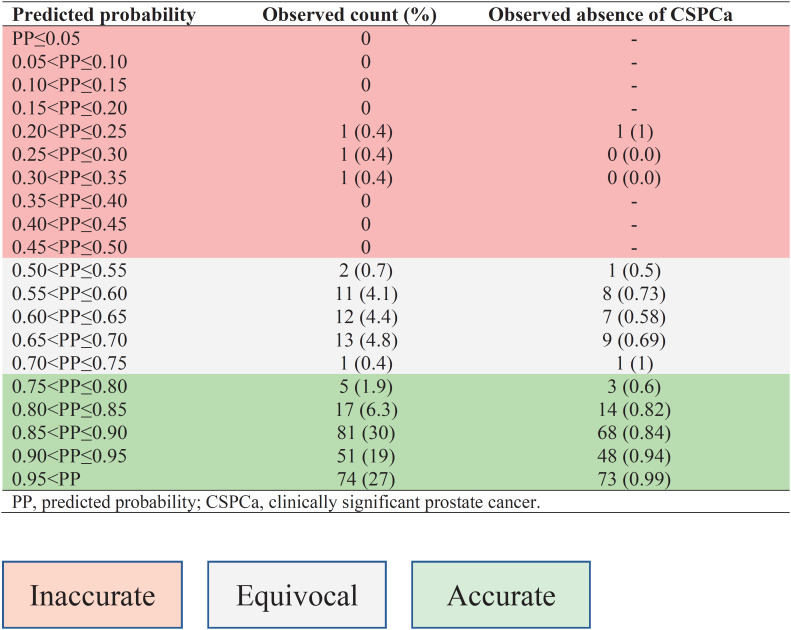

A. A 52-year-old Black man with PSA 8.5ng/mL and history of prior positive biopsy. Pre biopsy multiparametric MRI revealed no evidence for focal, high grade PCa. PV was 31mL and PSAD was 0.27ng/mL2. When the information is applied to the nomogram, the total points is 65 and PP of absence of CSPCa is 0.35. The PP is low and in the inaccurate range (red), therefore, systematic biopsy should be considered. Systematic biopsy revealed Grade Group 4 (Gleason score 4+4) prostate adenocarcinoma on right base to apex area including transitional zone.

B. A 73-year-old White man with PSA 8.5ng/mL and prostate biopsy naive. Pre-biopsy multiparametric MRI revealed no evidence for focal, high grade PCa. PV was 26mL and PSAD was 0.33ng/mL2. When the information is applied to the nomogram, the total points is 122 and PP of absence of CSPCa is 0.6. The PP is moderate and in the equivocal range (grey), therefore, systematic biopsy might be considered. Systematic biopsy revealed Grade Group 2 (Gleason score 3+4) prostate adenocarcinoma on right mid area. Grade Group 1 (Gleason score 3+3) prostate adenocarcinoma was detected on right base to apex area and left mid to apex area.

C. A 68-year-old White man with PSA 8.4ng/mL and history of prior negative biopsy. Pre biopsy multiparametric MRI revealed no evidence for focal, high grade PCa. PV was 92.8mL and PSAD was 0.09ng/mL2. When the information is applied to the nomogram, the total points is 310 and PP of absence of CSPCa is over 0.95. The PP is high and in the accurate range (green), therefore, the patient can safely avoid systematic PBx. Systematic biopsy confirmed benign histology.

T2W, T2 weighted; ADC, apparent diffusion coefficient; DWI, diffusion-weighted imaging; DCE, dynamic contrast-enhanced; MRI, Magnetic Resonance Image; PSA, Prostate specific antigen; PCa, prostate cancer; PV, prostate volume; PSAD, PSA density; PP, predicted probability; CSPCa, clinically significant PCa; PBx, prostate biopsy.

## DISCUSSION

A large systematic review and meta-analyses with a total of 42 studies including 7321 patients evaluated the negative predictive value of mpMRI and concluded that, regarding PBx, "local institutional data should form the basis of decision making if available" (22). The decision to perform PBx is multifactorial including: family history of PCa, race, history of prior biopsy, tumor markers, patient’s anxiety, etc. Many high-volume and reference centers selectively perform or do not perform PBx if mpMRI is classified PIRADS 1-2 (7, 23). In fact, there is unmet need of the method to predict the absence of CSPCa in males with negative MRI.

To facilitate personalized decision making without any intervention, we created a nomogram to predict the absence of CSPCa in males with PIRADS 1-2 mpMRI. The strengths/novelties of this study include: I) PBx were performed regardless of PIRADS classification; II) access to a prospectively maintained PBx database; III) all mpMRI followed current PIRADS standards at the time of PBx; IV) mpMRIs were reviewed by experienced radiologists; V) pragmatic sampling with sextant systematic biopsy; VI) inclusion of patients with different biopsy histories representing actual clinical practice; VII) inclusion of different races (minorities); VIII) no influence of any fusion system, since these were all non-targeted systematic biopsies; IX) no additional tests, other than those routinely used on clinical practice that are widely available PSA and PSAD; X) inclusion of mpMRI performed elsewhere allowing for wider use of the nomogram.

Our nomogram predicted the absence of CSPCa on PBx in males with PIRADS 1-2 mpMRI of the prostate using 327 consecutive patients with negative mpMRI. The selected model included age, black ethnicity, history of previous negative PBx, and PSAD ≥0.15ng/mL2 as predictors. Internal validation with 1,000x bootstrapping showed the fair discrimination performance (optimism corrected AUROC: 0.75). The current nomogram was further stratified into inaccurate, equivocal and accurate to deliver a clear information to users evaluating an individual probability of omitting PBx.

Other investigators have explored multifactorial chances of CSPCa on PBx in those with suspicious lesions on mpMRI (4, 24). Mehralivand et al. evaluated CSPCa (GG ≥2) detection using mpMRI and clinical variables, including race, in 400 patients with at least one mpMRI suspicious lesion (24). Different from Mehralivand et al, the current study focuses on patients without suspicious lesion on mpMRI.

Black males have increased risk of being diagnosed with PCa and more aggressive PCa (24, 25). Although, Black race is a predictor for CSPCa in males with PIRADS 3-5 mpMRI, to the best of our knowledge this study is the first to demonstrate that Black males with PIRADS 1-2 mpMRI also have increased risk for CSPCa on PBx (8, 22, 26). Some studies indicated higher aggressive PCa risk of Black males may be because of barriers to medical accessibility instead of genetic characteristics (27, 28). In our cohort, age, prostate volume, PSAD, and prior biopsy status were not significantly different between Black males and White males (Table-S2). Family history of PCa was also not significantly different; however, the difference was relatively large (47% vs 27%, p = 0.069). PSA, number of positive cores, the distribution of ISUP grade group, and maximum cancer core % were significantly different between the groups. Based on these results, Black males seemed to have higher risk of aggressive PCa at presentation. In some studies, age was an independent predictor for CSPCa on PBx (7, 29). In the current study, age was systematically selected via stepwise backward elimination method. Although age was not a significant predictor for CSPCa, we kept age in the nomogram because of its clinical relevance.

**Table S2:** Demographics of patients with negative MRI undergoing systematic prostate biopsy comparing Black and White males.

		Black	White	
Variables		Median (IQR) or N (%)	Median (IQR) or N (%)	p-value
No. of patients		19 (8)	214 (92)	–
Age, years		63 (59-69)	65 (59-70)	0.92
PSA, ng/mL		8.3 (4.4-11.1)	5.6 (4.1-8.1)	0.021
Prostate volume, mL		78 (31-137)	58 (39-86)	0.31
PSA density, ng/mL^2^		0.13 (0.07-0.21)	0.10 (0.06-0.14)	0.23
Suspicion DRE		5 (26)	30 (14)	0.18
Prior prostate biopsy status				0.36
Naive		4 (21)	70 (33)	
Prior negative		7 (37)	85 (40)	
Prior positive		8 (42)	59 (28)	
Family history prostate cancer		9 (47)	56 (27)	0.069
PIRADS version 2		14 (74)	129 (60)	0.33
No. of positive cores		1 (0-4)	0 (0-2)	0.0498
**ISUP Grade Group**				< 0.001
	0	8 (42)	135 (63)	
	1	3 (16)	54 (25)	
	2	5 (26)	16 (7)	
	3	1 (5)	8 (4)	
	4	1 (5)	0 (0)	
	5	1 (5)	1 (0.4)	
Maximum cancer core length, mm		6 (4-8)	3 (1-6)	0.075
Maximum cancer core, %		40 (10-60)	15 (5-30)	0.040

DRE = digital rectal examination; MRI = magnetic resonance image; IQR = interquartile range; PSA = prostate specific antigen; No. = number; PIRADS = Prostate Imaging-Reporting and Data System; ISUP = International Society of Urological Pathology.

The combination of PSAD and mpMRI has been investigated (4-9, 23, 29, 30). Pagniez et al. performed systematic review (16 studies) and meta-analyses (8 studies with 1,015 patients) and concluded that PSAD <0.15ng/mL2 in the presence of negative mpMRI was the most useful factor to identify males without CSPCa who could avoid PBx. However, they were unable to evaluate race (8). Similarly, we selected the PSAD ≥0.15ng/mL2 threshold because of its strong prognostication of CSPCa (Table-S3) (6, 8). If physicians use PSAD <0.15ng/mL2 alone for omitting systematic biopsy, 79.8% of systematic PBx for patients with negative MRI can be safely omitted, at the cost of missing 8.8% of CSPCa (Table-S4). The safely omittable systematic biopsy was 9% less than our nomogram using the cutoff of predicted probability 0.75. Regarding missed CSPCa, PSAD 0.15 cutoff is 0.8% less than predicted probability 0.75. Furthermore, to support decision-making, it is important to show how likely CSPCa will be detected. Thus, our nomogram is more useful than PSAD cutoff alone.

**Table S3:** Univariate analysis of PSAD as a continuous variable and a dichotomous variable.

		Univariate		
PSAD (ng/mL)		OR	CI (95%)	p-value
Continuous		0.0049	0.0002-0.12	0.001
**Dichotomous**				
	0.10	0.39	0.19-0.79	0.007
	0.11	0.30	0.15-0.61	<0.001
	0.12	0.28	0.14-0.56	<0.001
	0.13	0.34	0.17-0.67	0.002
	0.14	0.36	0.18-0.71	0.004
	0.15	0.28	0.14-0.56	<0.001
	0.16	0.30	0.15-0.61	0.001

CI = confidence interval; OR = odds ratio; PSAD = prostate specific antigen density.

**Table S4:** Subdivided performances of PSAD based on different cutoffs to predict the absence of CSPCa on prostate biopsy in males with negative MRI.

Cut off PSAD	Sensitivity	Specificity	NPV	PPV	Safely omittable PBx	Missed CSPCa
0.25	0.949	0.189	0.368	0.880	0.948	0.119
0.24	0.949	0.216	0.400	0.884	0.948	0.116
0.23	0.940	0.216	0.364	0.883	0.939	0.116
0.22	0.940	0.243	0.391	0.887	0.939	0.113
0.21	0.931	0.243	0.360	0.886	0.931	0.114
0.20	0.918	0.243	0.321	0.884	0.918	0.115
0.19	0.897	0.297	0.314	0.889	0.896	0.110
0.18	0.884	0.351	0.325	0.896	0.884	0.104
0.17	0.867	0.432	0.340	0.906	0.866	0.094
0.16	0.824	0.432	0.281	0.901	0.824	0.098
0.15	0.798	0.514	0.288	0.912	0.798	0.088
0.14	0.738	0.514	0.238	0.905	0.738	0.094
0.13	0.691	0.595	0.234	0.915	0.690	0.085
0.12	0.648	0.703	0.241	0.932	0.648	0.067
0.11	0.601	0.730	0.225	0.933	0.600	0.066
0.10	0.532	0.730	0.199	0.925	0.532	0.074
0.09	0.455	0.784	0.186	0.930	0.454	0.070
0.08	0.356	0.811	0.167	0.922	0.356	0.077
0.07	0.296	0.892	0.168	0.945	0.296	0.054
0.06	0.219	0.892	0.153	0.927	0.218	0.072
0.05	0.163	0.973	0.156	0.974	0.163	0.025

CSPCa = Clinically significant prostate cancer; NPV = negative predictive value; PBx = prostate biopsy; PPV = positive predictive value; PSAD = PSA density.

Safely omittable PBx was defined as dividing true positive cases by no CSPCa case number.

Missed CSPCa was defined as dividing false positive by case number bellow cutoff PSAD.

This study has limitations. This is a single center study with relatively small cohort. However, this is one of the largest American cohorts evaluating this specific population. Validation with 1,000x bootstrapping is a reasonable approach for such a cohort. Additionally, the nomogram showed a fair discrimination performance. Nevertheless, an external validation should be performed as a future work. The confidence interval was wide with low predicted probability in the "inaccurate" part of the nomogram. Therefore, we stratified and color-coded the nomogram on inaccurate, equivocal, and accurate to allow for straightforward interpretation by users. The accurate part of the nomogram with high prediction probability is useful for informed decision making about whether to skip PBx. Experienced radiologists at a tertiary referral center reviewed the MRIs; thus, the results may not have wide applicability. Nonetheless, external mpMRIs that satisfied the inclusion criteria were included. Twelve-core systematic biopsy as standard reference is less precise than saturation PBx. However, this is the standard of care in many centers. The data herein presented represents real world practice that we believe is applicable to daily practice.

## CONCLUSIONS

Our nomogram facilitates evaluation of individual probability of CSPCa on PBx in males with PIRADS 1-2 mpMRI and may be used to identify those in whom PBx may be safely avoided. Black race, history of previous negative PBx and PSAD ≥0.15ng/mL2 were independent predictors for CSPCa on PBx and included in the nomogram. This study also indicated that Black males may have increased risk of CSPCa on PBx, even in the setting of PIRADS 1-2 mpMRI.

## ABBREVIATIONS

ADC: apparent diffusion coefficient
AS: Active Surveillance
AUROC: Area under the receiver operating characteristic
CIPCa: Clinically insignificant prostate cancer
CSPCa: Clinically significant cancer
DCE: dynamic contrast-enhanced
DRE: digital rectal examination
DWI: diffusion-weighted imaging
GG: Grade Group
ISUP: International Society of Urological Pathology
mpMRI: Multiparametric MRI
MRI: magnetic resonance imaging
PBx: prostate biopsy
PCa: prostate cancer
PIRADS: Prostate Imaging-Reporting and Data System
PP: Predicted probability
PSA: Prostate specific antigen
PSAD: Prostate specific antigen density
PV: Prostate volume
ROC: receiver operating characteristic
T2W: T2-weighted
TRUS: Transrectal ultrasound
USC: University of Southern California
